# The role of social determinants on tuberculosis/HIV co-infection mortality in southwest Ethiopia: a retrospective cohort study

**DOI:** 10.1186/s13104-016-1905-x

**Published:** 2016-02-12

**Authors:** Hailay Gesesew, Birtukan Tsehaineh, Desalegn Massa, Amanuel Tesfay, Hafte Kahsay, Lillian Mwanri

**Affiliations:** Department of Epidemiology, College of Health Sciences, Jimma University, Jimma, Ethiopia; School of Statistics and Mathematics, Faculty of Science, Alberta University, Edmonton, Canada; Department of Population and Family Health, Jimma University, Jimma, Ethiopia; ART Clinic, Filtu Hospital, Somali, Ethiopia; Discipline of Public Health, Faculty of Medicine, Nursing and Health Sciences, Flinders University, Adelaide, Australia

**Keywords:** Tb/HIV, Co-infection, Social determinants, Retrospective cohort, Ethiopia

## Abstract

**Background:**

The role played by social determinants of health including social, economic, environmental and cultural factors in influencing health outcomes for many health conditions has been widely described. However, the potential impact of these factors on morbidity and mortality of infectious diseases particularly tuberculosis (Tb)/HIV co-infection mortality is scantly addressed. We assessed the role that social determinants play in Tb/HIV co-infection mortality in southwest Ethiopia.

**Methods:**

A retrospective cohort study collated Tb and HIV data from Jimma University Teaching Hospital, Southwest Ethiopia for the period of September 2010 and August 2012. Data analysis was conducted using STATA version 14 for mackintosh. Both descriptive and inferential statistics analyses were performed. Logistic regression was applied to identify factors associated with Tb/HIV co-infection mortality at P value of ≤0.05 in the final model.

**Results:**

Fifty-five (20.2 %) patients died during the study period. Compared to their counterparts, more Tb/HIV co-infection death was observed in young age groups between 25 and 34 years (47.3 %), females (58.2 %), daily labors (40 %) and Muslim followers (54.5 %). 43.6 and 41.8 % of study participants respectively had single and double bedrooms, and 25.5 and 23.6 % of deceased study participants did not have water and electricity in the household respectively. Logistic regression analyses demonstrated the following factors significantly associated with Tb/HIV co-infection mortality: being a commercial sex worker (AOR, 5.6; 95 % CI, 1.2–25.8), being of bed ridden functional status (AOR, 3.9; 95 % CI, 1.5–10.3) and being a rural resident (AOR, 3.4; 95 % CI, 1.4–8.4).

**Conclusions:**

One-fifth of Tb/HIV co-infected patients died due to the co-infection. Social determinants including type of occupation, severity of disease and residing in rural areas seemed to have a significant association with the poor disease outcome. Findings of this study inform the role that social determinants play in influencing mortality due to Tb/HIV co-infection. Consistent with principles of primary health care as stated by Alma Ata declaration, and in order to achieve better disease outcomes, intervention frameworks that address Tb/HIV mortality should not only focus on the medical interventions of diseases, but should also integrate and improve social determinants of affected populations.

## Background

Social determinants of health have broadly been defined as social, economic, cultural and environmental factors and circumstances that influence health outcomes of populations [1, 2]. These factors influence health at different levels including, at individual behavioral level, health care system level and the surrounding environmental level [3]. The factors may include education, religion, employment, income & job security, housing, food security, poverty, health services and access to services, inequalities, social exclusion, stigma and cultural issues [3].

Despite nearly four decades since the Alma Ata declaration and the recognition of the necessity of primary health care in providing basic health care, the current health systems across the world do not adequately address elements of social determinants of health in planning for health interventions and in the management of health conditions [4, 5]. Recognizing this setback, the World Health Organization (WHO) established the Commission on Social Determinants of Health (CSDH) in 2005 to highlight the importance of causes of poor health outcomes and inequalities across the globe, across countries and within countries [1, 6].

It is a common knowledge that the above listed social determinants of health influence both infectious and non-infectious diseases especially in poor communities [7, 8]. Tuberculosis (Tb) and human immunodeficiency virus (HIV) are the most important infectious diseases of our time. In 2013, Ethiopia is a country with high TB burden, high HIV burden and high MDR-Tb (multi-drug resistance Tb) [9]. The country ranked third in Africa and eighth among the highest Tb burden countries in the world [10]. Tb and HIV have had severe negative socioeconomic, cultural and environmental impacts across developing countries [2, 11]. Tb/HIV co-infection causes serious bidirectional effect than either of the two diseases alone in sinking the effect of antiretroviral therapy (ART) and short course directly observed treatment (DOTS) [12].

Studies that have described the association between social determinants and co-existence of Tb/HIV illnesses are scarce. However, a study conducted in south Asia informed that poverty, food insecurity, malnutrition, religious and cultural issues were the key determinants of Tb/HIV co-infection [3]. In addition, addressing gender, racial, age, marital and ethnic inequalities have been noted to be important for the success of Tb/HIV co-infection management outcomes [13–15].

Previous studies have focused on describing the social determinants and Tb/HIV co-infection only but their relationship with the outcome of the co-infection is not addressed. The current study examined the association between social determinants of health and Tb/HIV co-infection mortality.

## Methods

### Study design, setting and participants

A retrospective cohort study using records from September 01, 2010 to August 31, 2012 was carried out in ART clinic at Jimma University Teaching Hospital (JUTH), southwest Ethiopia. JUTH is located in Jimma zone, 357 km southwest of Addis Ababa, the capital city of Ethiopia and its catchment area comprises a population of 2486,155 people of which 89.69 % are rural inhabitants [16]. An average of 4.8 persons to a household (3.8 in urban and 5 in rural households) are residing in the district [16]. Jimma is found in Oromia region, a region that accounted for the highest number of HIV infected people from Ethiopia. It is near Gambella region, a region that accounted for the highest prevalence rate of HIV from Ethiopia [17]. The city is also near a refugee camp where a number of refugees from different African countries flee in. The presence of high emigration and immigration from and to the city put at risk to both Tb and HIV. Primary healthcare services, including diagnosis and treatment of Tb, voluntary counseling and testing (VCT), prevention of mother to child transmission (PMTCT), ART and opportunistic infections (OIs) treatment services are available. Data were extracted between August and October 2013. All patients aged ≥15 years and who had access to Tb/HIV medical treatment at JUTH were the target population. Incomplete records were excluded from the analysis.

### Data source

A data extraction checklist was used to extract information from a database called electronic medical records or EMR system designed since 2007, a system also called comprehensive care centre patient application database (C-PAD). Patient information was recorded by clinicians on paper forms, the paper based system, followed by data entry into EMR by data clerks on the same day as the clinic visit or the next day. The patient information included clinical and non-clinical characteristics of the patient. To enter the data, if all patient information is available, it takes about 10 min for new patient and about 5 min for revisit patient. Data clerks make immediate communication with clinicians when any data is missed, and weekly EMR-generated patient summary reports help to flag patients with conditions that seek follow-up. When data were incomplete, we tried to refer the patient cards, registration and log books.

### Variables of the study and measurement

Tb/HIV co-infection outcome was dichotomized to death and alive as the dependent variable. The explanatory variables included age, religion, educational level, marital status, occupation, residence, number of bedrooms, past opportunistic infection, Tb incidence, functional status, baseline CD4^+^ T cells count (CD4 cells count), baseline weight and WHO clinical stage. Level of education was classified as illiterate (couldn’t read and write), read and write only (could read and write but received no formal education) and formal education (received formal education starting from grade one). Functional status was categorized as work (able to perform usual work), ambulatory (able to perform activity of daily living) and bedridden (not able to perform activity of daily living).

### Statistical analysis

Data exploration, editing and cleaning were undertaken before analysis. The analysis of both descriptive and inferential statistics was conducted. Descriptive statistics included mean, median, standard deviations and range values for continuous data; percentage and frequency tables for categorical data. Logistic regression was used to identify factors associated with Tb/HIV co-infection mortality. Bivariate logistic regression analysis was conducted to see the existence of crude association and select candidate variables (with P value below 0.25 were considered) to multivariable logistic regression. We checked multi-collinearity among selected independent variables via variance inflation factor (VIF) and none was found. *P* value ≤0.05 was considered as a cut point for statistical significance in the final model. Fitness of goodness of the final model was checked by Hosmer and Lemeshow test and was found fit. The Data was summarized using odds ratio (OR) and 95 % confidence interval. Data analysis was conducted using STATA version 14 for mackintosh.

### Ethics statement

Ethical clearance was obtained from the office of institutional ethical review board (IRB) of College of Health sciences, Jimma University. Waiver of the consent was obtained from IRB of JUTH. The data access permission was obtained from JUTH board. No participant was actually involved in the study—we did simply extract anonymised data from the record. The data and collected information was kept and locked in a filing cabinet with the key only accessible to principal investigator (PI) and the computer files was protected with passwords that only the PI knows.

## Results

### Socio-demographic and economic characteristics of the all study participants

**Fig. 1:**
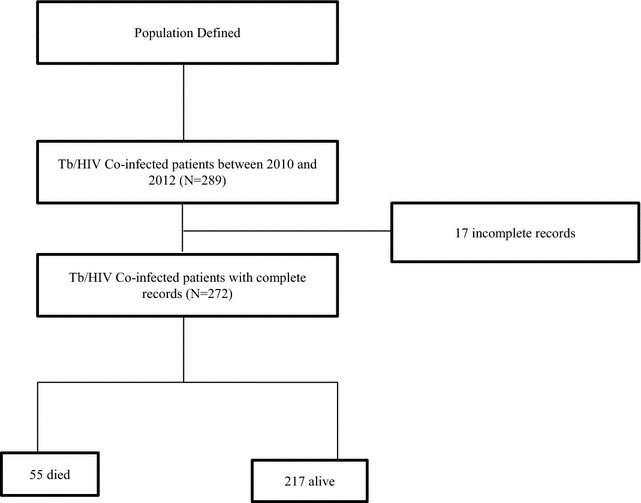
Schematic presentation of data extraction of Tb/HIV co-infection mortality among Tb/HIV co-infection patients

During the period between September 2010 and August 2012, 289 patients were registered for Tb treatment in JUTH (Fig. 1), whereof 17 records were incomplete in all data sources. In total, complete records of 272 Tb/HIV co-infected patients were included for analysis. Table 1 shows demographic and economic characteristics of the respondents. Majority of the study participants were between 25 and 34 years with a mean age of 32(±8.53) years, and females accounted for nearly half (49.3 %) of the study participants. Muslim followers were over-represented (58.1 %) and one-third (31.6 %) of participants represented daily laborers. Nearly half (51.5 %) of the patients attained formal education and two-third (60.7 %) of the respondents were married. Compared to rural settlers, urban dwellers comprised a significant proportion (70 %) of the study participants.

**Table 1:** Socio-demographic and economic characteristics of Tb/HIV co-infected patients at JUTH, southwest Ethiopia, 2013

Variable	Category	Number (n = 272)	Percent
Age (in years)	15–24	38	13.9
	25–34	140	51.5
	35–44	69	25.4
	≥45	25	9.2
	Mean age	32 (±8.53) years	
Sex	Male	114	41.9
	Female	158	58.1
Occupation	Government employed	48	17.7
	NGO	46	16.9
	Farmer	80	29.4
	Daily labor	86	31.6
	Commercial sex worker	12	4.4
Educational status	Illiterate	81	29.8
	Read and write (informal)	51	18.8
	Formal education	140	51.4
Religion	Orthodox	76	27.9
	Muslim	134	49.3
	Protestant	50	18.4
	Catholic	12	4.4
Marital status	Married	165	60.7
	Single	58	21.3
	Divorced	36	13.2
	Widowed	13	4.8
Residence	Urban	189	69.5
	Rural	83	30.5

### Demographic and social characteristics of the deceased participants

Table 2 shows demographic and social characteristics of the deceased participants. The prevalence of Tb/HIV co-infection mortality was registered among 55 (20.2 %) study participants. Of these, participants aged between 25–34 years and 35–44 years accounted for 47.3 and 38.2 % of Tb/HIV co-infection mortality respectively. Females contributed more than half (58.2 %) of Tb/HIV co-infection mortality. The mortality was also higher among married (60.1 %) compared to single (23.6 %) study participants. When analyzed according to religion, Muslim followers accounted for 54.5 % of mortality whereas catholic followers were the least contributors to mortality and accounted for 7.3 %. Tb/HIV co-infection mortality was very high among occupants with economic hardship. Daily laborers and farmers contributed to 40 and 20 % of mortality in the study participants respectively. Just over 40 % of the deceased Tb/HIV co-infected study participants were formally educated whereas 36 % were illiterate. The remaining deceased participants were recorded as being able to read and write but did not receive formal education.

**Table 2:** Social determinants of Tb/HIV co-infection mortality at JUTH, southwest Ethiopia, 2013

Variable	Category	Tb/HIV co-infection status (n = 272)	
		Alive, n (%)	Died, n (%)
Age (in years)	15–24	32 (14.7)	6 (10.9)
	25–34	114 (52.5)	26 (47.3)
	35–44	48 (22.1)	21 (38.2)
	≥45	23 (10.7)	2 (3.6)
Sex	Male	91 (41.9)	23 (41.8)
	Female	126 (58.1)	32 (58.2)
Occupation	Government employee	40 (18.4)	8 (14.5)
	NGO	37 (17.1)	9 (16.4)
	Farmer	69 (31.8)	11 (20)
	Daily labor	64 (29.5)	22 (40)
	Commercial sex worker	7 (3.2)	5 (9.1)
Educational status	Illiterate	61 (28.1)	20 (36.4)
	Read and write (informal)	39 (18)	12 (21.8)
	Formal education	117 (53.9)	23 (41.8)
Religion	Orthodox	65 (30)	11 (20)
	Muslim	104 (47.9)	30 (54.5)
	Protestant	40 (18.4)	10 (18.2)
	Catholic	8 (3.7)	4 (7.3)
Marital status	Single	45 (20.7)	13 (23.6)
	Married	132 (60.9)	33 (60.1)
	Divorced	28 (12.9)	8 (14.5)
	Widowed	12 (5.5)	1 (1.8)
Residence	Urban	154 (71)	35 (63.6)
	Rural	63 (29)	20 (36.4)
Number of people living with	<5	172 (79.3)	38 (69.1)
	≥5	45 (20.7)	17 (30.9)
Water	Yes	166 (76.5)	41 (74.5)
	No	51 (23.5)	14 (25.5)
Electricity	Yes	165 (76)	42 (76.4)
	No	52 (24)	13 (23.6)
Room	1	97 (44.7)	24 (43.6)
	2	87 (40.1)	23 (41.8)
	3	24 (11.1)	7 (12.7)
	4	9 (4.1)	1 (1.9)
Functional status	Work	112 (51.6)	26 (47.3)
	Ambulatory	79 (36.4)	16 (29.1)
	Bed ridden	26 (12)	13 (23.6)
WHO stage	1	11 (5.1)	7 (12.7)
	2	55 (25.3)	12 (21.8)
	3	101 (46.5)	23 (41.8)
	4	50 (23.1)	13 (23.7)
Tb type	Pulmonary	172 (79.3)	41 (74.5)
	Extra pulmonary	11 (5.1)	3 (5.5)

Great majority (63.6 %) of deceased were urban dwellers. A significant proportion (30.9 %) of deceased lived with more than five individuals in a single house. Among the deceased, 24 (43.6) and 23(41.8 %) of study participants had single and double bedrooms respectively; where as those with four bedrooms contributed the least (1.9 %) to Tb/HIV co-infection mortality. Regarding water and electric availability, 25.5 and 23.6 % of deceased study participants did not have water and electricity in their households. Over one-fifth (23.6 %) deceased study participants were recorded as seriously ill and bed ridden. Majority of deceased study participants had pulmonary Tb type (74.5 %) followed by mixed type (20 %).

### Social determinants associated with Tb/HIV co-infection mortality

Factors associated with the Tb/HIV co-infection mortality were analyzed. Age, sex, educational status, occupational status, patient referral status, number of people per house hold, functional status, weight, WHO stage and Cd4 count had p value ≤0.25 in bivariate logistic regression and were candidates for multiple logistic regression.

Table 3 presents the multiple logistic regression analysis with social determinants of health and Tb/HIV co-infection mortality. Logistic regression analyses demonstrated the following factors associated with Tb/HIV mortality: being a commercial sex worker, being a rural resident and being recorded as bed-ridden functional status. Commercial sex workers were nearly 6 times (AOR, 5.6; 95 % CI, 1.2–25.8) higher at risk of death than government employee. The relative probability of Tb/HIV co-infection mortality among those with bed-ridden status was higher than (AOR, 3.9; 95 % CI, 1.5–10.3) with work status. The association of Tb/HIV mortality among rural residents was 3.4 times (AOR, 3.4; 95 % CI, 1.4–8.4) higher than among those who were urban residents.

**Table 3:** Multiple logistic regression predictors of Tb/HIV co-infection mortality at JUTH, southwest Ethiopia, 2013

Variable	Category	Tb/HIV co-infection status (n = 272)		Odds ratio	
		Alive, n (%)	Died, n (%)	Crude OR (95 % CI)	Adjusted OR (95 % CI)
Age (in years)	15–24	32 (84.2)	6 (15.8)	1	1
	25–34	114 (81.4)	26 (18.6)	1.2 (0.4–3.2)	0.9 (0.3–2.8)
	35–44	48 (69.6)	21 (30.4)	2.3 (0.9–6.4)	1.8 (0.6–5.8)
	≥45	23 (92)	2 (8)	0.5 (0.1–2.5)	0.3 (0.05–1.8)
Occupation	Government employee	40 (83.3)	8 (16.7)	1	1
	NGO	37 (80.4)	9 (19.6)	1.2 (0.4–3.5)	1.02 (0.3–3.2)
	Farmer	69 (86.2)	11 (13.8)	0.8 (0.3–2.2)	0.3 (0.08–1.1)
	Daily labor	64 (74.4)	22 (25.6)	1.7 (0.7–4.2)	1.4 (0.5 –3.8)
	Commercial sex worker	7 (58.3)	5 (41.7)	3.6 (0.9–14.1)	*5.6* (*1.2–25.8* ) ^a^
Residence	Urban	154 (81.5)	35 (18.5)	1	1
	Rural	63 (75.9)	20 (24.1)	1.4 (0.8–2.6)	*3.4* (*1.4–8.4*)^a^
Functional status	Work	112 (81.2)	26 (18.8)	1	1
	Ambulatory	79 (83.2)	16 (16.8)	0.9 (0.4–1.7)	1.3 (0.6–2.9)
	Bedridden	26 (66.7)	13 (33.3)	2.2 (0.9–4.7)	*3.9* (*1.5–10.3*)^a^

^a^Denotes statistically significant in final model at P value of ≤0.05 in the final model

## Discussion

It is well recognized that health outcomes of populations are remarkably influenced by complex social, cultural, environment and economic systems briefly termed as social determinants of health [1, 3, 18, 19]. This study examined the association between social determinants and Tb/HIV co-infection mortality.

Results of this study showed that one out of five Tb/HIV co-infected patients were deceased. This finding was similar to the WHO reports that have shown same findings whereof Tb has contributed (26 %) for HIV mortality elsewhere [20]. Comparable findings were described in Ethiopia [21] and Malaysia [22]. Consistent with the findings of similar studies from Zambia [15] and Spain [23], the current study showed that Tb/HIV co-infection mortality was highly present among young age group people between 25 and 34 years. This age group which is the most productive in any population and setting, has also been reported to have the highest burden of HIV [15]. The mortality was also registered among females than males, a finding similar to the study conducted in Zambia [15]. A review from south Asia also reported that gender inequality added fuel to HIV epidemic [3], calling for an integrative approach to address HIV and reduce sequel among young age groups and females. This will have a policy implication on the integrated approach in reducing the risky sexual behaviour particularly young age groups and females.

In the current study, Tb/HIV co-infection mortality was high among participants with economic hardships including those with occupations such as daily laborers (who earn very little), indicating that Tb/HIV co-infection mortality was high among the poor. Poverty has been acknowledged as a significant determinant of health in many settings across the globe [2, 11, 18]. Patients recorded as being bedridden carried a higher risk of death compared to when being of work status. These findings support studies conducted in other parts of Ethiopia including in Hawassa University Teaching Hospital [24] and Ambo Referral Hospital [25].The current study also reported that 36.4 % of deceased study participants were illiterate, a finding supported by Zambian study [15]. Poor education has been mentioned in many studies across the globe to be a significant social determinant of poor health and inequity in many health conditions [2, 11, 18, 26–28]. Hence, the Ministry of Health and Ministry of Education together with other sectors should design a tailored strategy to halt the tides of Tb/HIV co-infection mortality.

Gender inequality, occupation disadvantage and education status were also found to be predictors of mortality due to Tb/HIV co-infection, affirming the complex nature and the contribution of socio-cultural and economic factors to disease outcomes [2, 11].

Tb/HIV co-infection mortality was also influenced by the setting of participant with rural dwellers being more likely to die compared to their urban counter parts. This finding was not a surprise as has been reported elsewhere and attributed to factors such as accessibility of the health care services and transportation availability [1, 29]. Significant proportion of deceased study participants lived with more than five individuals in a single house, had single and double bedrooms, and lacked water and electricity supply. It is plausible therefore to hypothesise that overcrowding and lack of water leading to poor sanitation could be significant factors that exacerbate conditions especially Tb in poor settings. These factors have also been supported by several studies conducted across the globe [23, 30, 31]. This cues the need of revising intervention framework of Tb/HIV mortality that was focused on health sector alone. Efforts should be made to integrate and improve prevailing social determinants in a particular setting.

As to functional status is concerned, bedridden patients had four times (AOR, 3.9, 95 % CI: 1.5–10.3) an increased risk of mortality than in working status. This not dissimilar with the study conducted in Bahir Dar [21] that reported bedridden patients were nearly four times at risk of dying than in work functional status (AHR = 3.88; 95 % CI: 2.15–7.02). Other studies [32–34] also supported the evidence.

This poor baseline performance scale might be due late presentation for HIV care, a big challenge in the HIV care continuum. We recommend further works in this.

Worth noting limitations should have noted in this study. First, information of some variables was incomplete and rejected from analysis even though we checked variety data sources. Second, the sample was small due to the short follow up period. Third, variables that could potentially have confounding effects such as alcohol abuse, occupation, illicit drug use, drug resistance, co-morbidities, treatment adherence and severity of immune suppression were not measured. Besides, HIV related stigma, cultural preferences, food insecurity, inequalities, deprivation and malnutrition were also not measured due to the nature of the design.

## Conclusion

In summary, the findings from the current study agree in many points with the findings of previous publications. Findings of the current study indicated that one-fifth of Tb/HIV co-infected patients were deceased and social factors seemed to have significant influence. Even though there are current effective medical treatments for Tb/HIV co-infection, which are well supported by WHO policies, it is plausible to suggest that intervention framework of Tb/HIV mortality should not solely be addressed from the health sector alone. Efforts should be made to integrate and improve prevailing social determinants in a particular setting. We recommend further research to consider the role of other potential social determinants including stigma, cultural preferences, food insecurity, inequalities, deprivation and malnutrition. Further studies with multilevel analysis to simultaneously explore the influence of individual and contextual factors would also provide additional evidence.

## Abbreviations

WHO: World Health Organization
CSDH: commission on social determinants of health
Tb: tuberculosis
HIV: human Immunodeficiency Virus
MDR-Tb: multi-drug resistance Tb
ART: antiretroviral therapy
JUTH: Jimma University Teaching Hospital
VCT: voluntary counseling and testing
PMTCT: prevention of mother to child transmission
EMR: electronic medical records
C-PAD: comprehensive care centre patient application database
VIF: variance inflation factor
